# Femtosecond electronic structure response to high intensity XFEL pulses probed by iron X-ray emission spectroscopy

**DOI:** 10.1038/s41598-020-74003-1

**Published:** 2020-10-08

**Authors:** Roberto Alonso-Mori, Dimosthenis Sokaras, Marco Cammarata, Yuantao Ding, Yiping Feng, David Fritz, Kelly J. Gaffney, Jerome Hastings, Chi-Chang Kao, Henrik T. Lemke, Timothy Maxwell, Aymeric Robert, Andreas Schropp, Frank Seiboth, Marcin Sikorski, Sanghoon Song, Tsu-Chien Weng, Wenkai Zhang, Siegfried Glenzer, Uwe Bergmann, Diling Zhu

**Affiliations:** 1 SLAC National Accelerator Laboratory, Menlo Park, CA 94025 USA; 2 Univ Rennes, CNRS, IPR (Institut de Physique de Rennes), UMR 6251, 35000 Rennes, France; 3 SwissFEL, Paul Scherrer Institute, 5232 Villigen, Switzerland; 4 Deutsches Elektronen-Synchrotron DESY, Notkestraße 85, 22607 Hamburg, Germany; 5 European XFEL, Holzkoppel 4, 22869 Schenefeld, Germany; 6 School of Physical Science and Technology, ShanghaiTech University, Shanghai, 201210 China; 7 Department of Physics and Applied Optics Beijing Area Major Laboratory, Center for Advanced Quantum Studies, Beijing Normal University, Beijing, 100875 China

**Keywords:** Chemistry, Physical chemistry

## Abstract

We report the time-resolved femtosecond evolution of the K-shell X-ray emission spectra of iron during high intensity illumination of X-rays in a micron-sized focused hard X-ray free electron laser (XFEL) beam. Detailed pulse length dependent measurements revealed that rapid spectral energy shift and broadening started within the first 10 fs of the X-ray illumination at intensity levels between 10^17^ and 10^18^ W cm^-2^. We attribute these spectral changes to the rapid evolution of high-density photoelectron mediated secondary collisional ionization processes upon the absorption of the incident XFEL radiation. These fast electronic processes, occurring at timescales well within the typical XFEL pulse durations (i.e., tens of fs), set the boundary conditions of the pulse intensity and sample parameters where the widely-accepted ‘probe-before-destroy’ measurement strategy can be adopted for electronic-structure related XFEL experiments.

## Introduction

The seminal work by Neutze et al. in 2000^1^ evaluated for the first time the timescale of the Coulomb explosion of a protein molecule when illuminated by an intense X-ray pulse. This set the basic concept that protein structure determination can be achieved if sufficiently short and intense X-ray pulses are used. This ‘probe-before-destroy’ approach has been a cornerstone measurement strategy for many pioneering experiments using X-ray free electron lasers (XFELs) in many areas including single particle imaging^2,3^, serial femtosecond crystallography^4,5^, coherent diffractive imaging^6^ and ultrafast X-ray spectroscopy^7–12^. It was demonstrated for serial femtosecond crystallography at Å resolution, that an X-ray probe pulse length on the order of tens of femtoseconds can ‘outrun’ the anticipated sample damage caused by an intense X-ray pulse containing 10^11^–10^12^ photons focused down to a few μm^13,24^. Recent diffraction and spectroscopy data using micron-sized focused XFEL beams with intensity levels reaching 10^17^–10^18^ W cm^-2^ have indicated that such intensities could trigger various damage mechanisms impacting experimental observables within the typical X-ray pulse durations of 10–30 fs^14–17^. In particular some efforts have been made to model and predict spectral shifts and tolerable photon densities for solution phase experiments^17^, and to describe the effect of solvent photoionization on the spectral shape^16^. In this letter we report a systematic time-dependent study of the effects of high XFEL pulse intensity on the electronic structure of Fe compounds at various metal concentration probed by X-ray emission spectroscopy (XES). Our results provide not only a baseline for the beam parameters of XFEL-based XES measurements on transition metal systems, but also key experimental data for calibrating numerical models for ultrafast X-ray matter interactions.

K-shell X-ray emission spectroscopy (XES) is a local electronic structure probe primarily used for 3d transition metals, where the core-level radiative decay (the X-ray fluorescence) occurs within ∼1 fs upon vacancy formation^18^. XES can be separated into core-to-core (CTC) XES (Kα, Kβ_1,3_, Kβ’) and valence-to-core (VTC) XES (Kβ_2,5_, Kβ’’), each with specific sensitivity to the instantaneous local electronic structures of the absorbing atoms including details of their oxidation and spin states, covalency and ligand environments^18^. If rearrangement of the electronic structure occurs to a significant fraction of atoms being probed during the duration of an XFEL pulse, the XES signal from the thereby modified atoms may contribute to the observed XES signal. In this letter, we report a systematic study of the time-resolved femtosecond-scale evolution of the K-shell XES spectra of Fe when illuminated with intense XFEL pulses. We observe initiation of spectral energy shifts and broadening of the CTC-XES well within the first 10 fs of X-ray illumination at intensity levels ranging from 10^17^ to 10^18^ Wcm^−2^. The VTC-XES intensity starts to decrease essentially instantaneously (< 4 fs) and eventually results in a complete peak loss at times > 15 fs. We find that these effects are strongly dependent on the XFEL photon flux, pulse duration and concentration/density of the dominant absorbing element.

## Results and discussion

**Figure 1:**
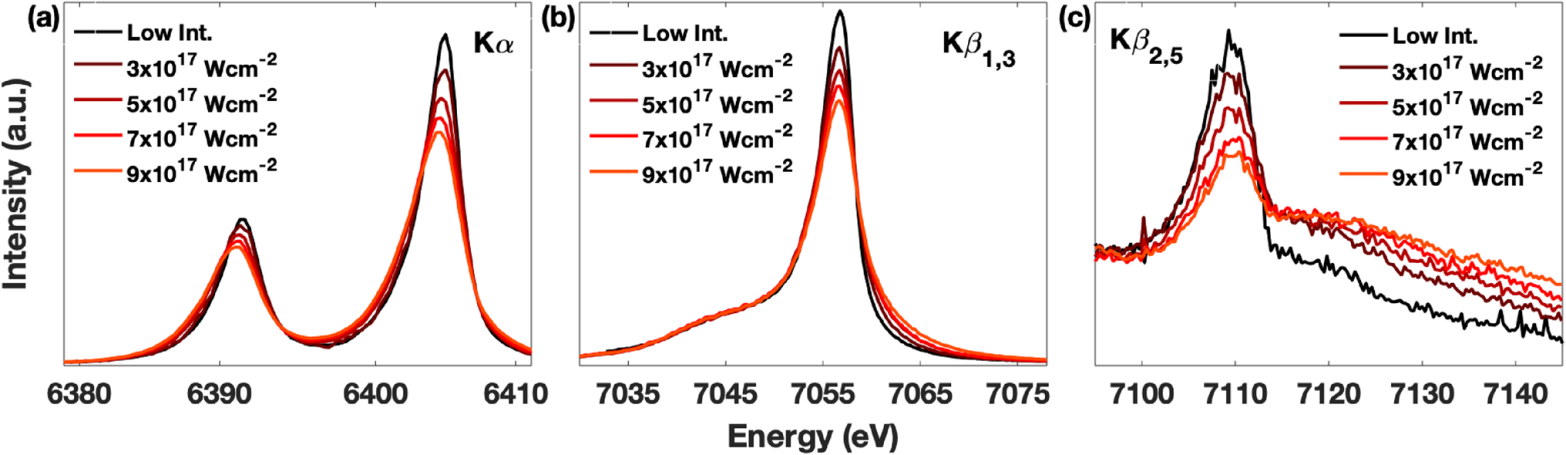
Fe emission spectra **(a)** Kα, **(b)** Kβ_1,3_, and **(c)** Kβ_2,5_ measured with 10 fs X-ray pulses at different intensity levels up to ~ 9 × 10^17^ W cm^−2^. All spectra are normalized to the shot-to-shot measurement of the incoming pulse energy.

Figure 1 shows the XES spectra of metallic Fe as a function of intensity for a constant pulse length of 10 fs. The most intense pulses correspond to a pulse energy of ∼0.4 mJ with a 2 μm diameter focal spot on the sample (~ 9 × 10^17^ W cm^−2^). Notable spectral changes are observed. Both Kα and Kβ_1,3_/Kβ’ spectra show a gradual broadening as a function of intensity (Fig. 1a,b). While the Kα spectra show a slight shift to lower energies, the most prominent spectral shape change in Kβ_1,3_ is the redistribution of spectral weights towards the high energy tail. The most dramatic changes are seen in the Kβ_2,5_ spectra, where the overall and peak intensities strongly decrease, and the spectrum widens to higher energies (Fig. 1c).

**Figure 2:**
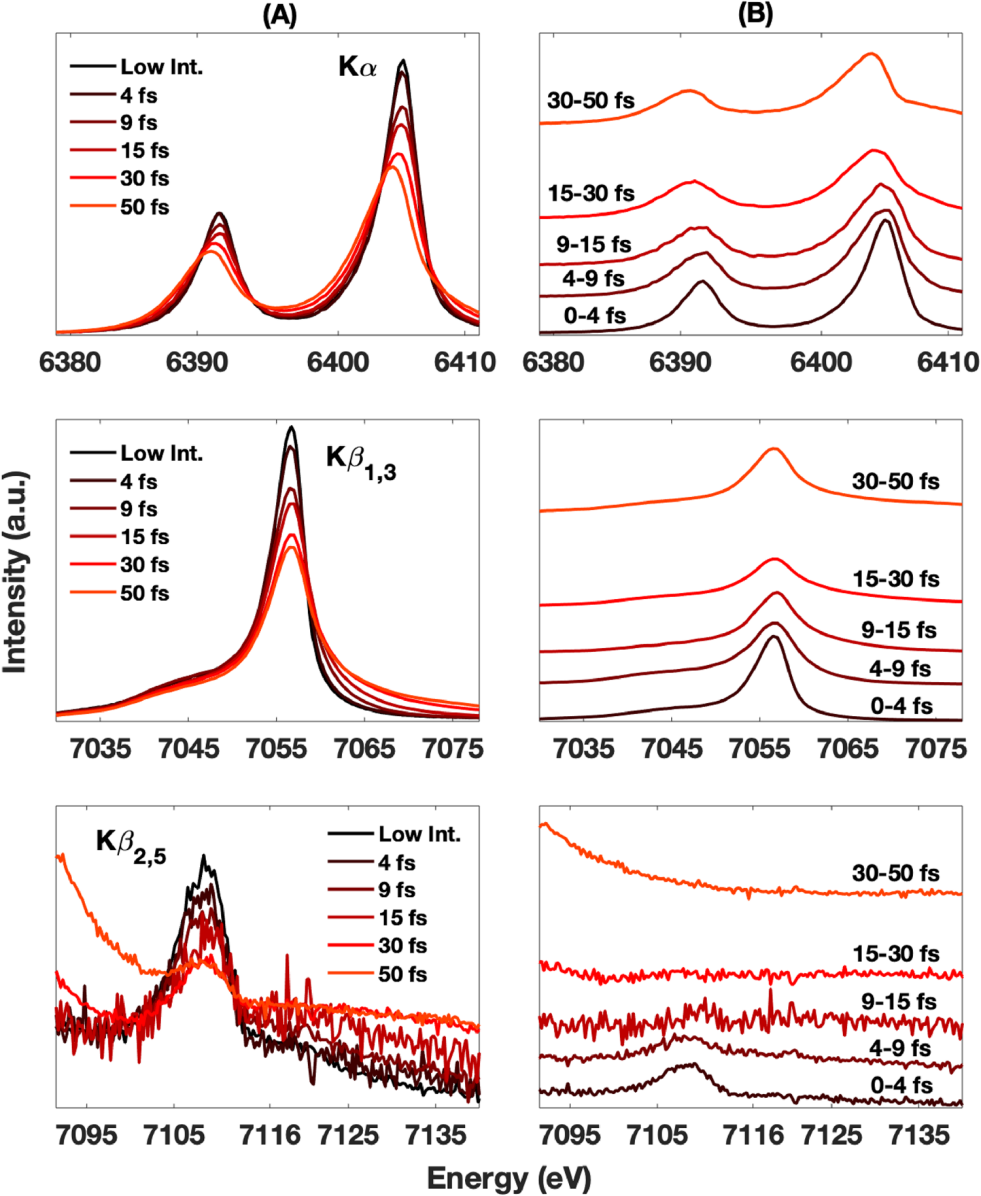
Column A shows the Kα, Kβ_1,3_, and Kβ_2,5_ cumulative emission spectra (top, middle, bottom respectively) measured with different X-ray pulse durations at an average intensity of 3 × 10^17^ W cm^-2^. Their respective deconvolved spectral evolution are shown on column B. The deconvolved traces are vertically offset by the amounts proportional to their timing.

Figure 2A shows the XES spectra of metallic Fe as a function of pulse lengths ranging from 4 to 50 fs, at a constant intensity (average of ∼3 × 10^17^ W cm^−2^). Sub 10 fs resolution spectral evolution can be extracted from the measured integrated XES spectra by weighted subtractions of the earlier time contributions from the later ones. For example, spectra are measured with 4 fs and 9 fs pulses respectively. By subtracting the weighted 4 fs spectrum from the 9 fs spectrum, one obtains a spectrum corresponding to how the system has evolved in the 5–9 fs time window. Figure 2B shows the XES spectra obtained with this method. The integrated spectra show similar but more pronounced trends as compared to those in Fig. 1. Specifically, the shift of the Kα lines to lower energies is more pronounced, as clearly seen in the shifted peak positions (Fig. 2A top). This trend is even more prominent on Fig. 2B top, where we show extracted Kα spectra corresponding to different time periods. As in Fig. 1, the Kβ_1,3_ spectra show a gradual broadening, mainly to the higher energy side, as a function of pulse length. However, no notable shift of the spectral maximum is observed, in contrast with the Kα spectra. As in Fig. 1, the Kβ_2,5_ spectral changes are the strongest, leading to a complete intensity loss after about ∼9 fs (Fig. 2B bottom).

At the intensity level of this experiment, the probability of sequential double-absorption, i.e., an individual atom undergoing photoabsorption twice within the pulse duration, is rather low. For a 0.4 mJ (at the sample, considering all beam transport losses), 8.2 keV, 10 fs X-ray pulse focused down to 2.0 μm diameter, the average intensity is approximately 3 × 10^17^ W cm^−2^, and the probability of double-absorption within the pulse length is only about 2.9%. During this experiment we observed very weak Kα hypersatellite lines, a direct indication of double ionization^19^, which is consistent with this few percent estimate. Thus, we believe that the dominant physical processes that lead to the observed large spectral alterations are most likely related to secondary processes of electron–electron and electron–ion collisions. This results in the photoabsorption by atoms that are ionized by the electron cascade created earlier within the X-ray pulse. Note that the energy deposited per Fe atom in our measurements was about 200 eV, which is comparable with the energy necessary to remove three 3d and two 4s electrons. The energy shift of the Kα lines and the spectral weight redistribution of the Kβ lines can be qualitatively explained via configuration-average Hartree–Fock calculations^20^ in the isolated-atom approximation.

Reproducing the detailed X-ray emission shape requires advanced approaches to account for the spin–orbit coupling (e.g. Kα_1_ vs Kα_2_), the exchange interaction and the multiplet effects^21^. Accurate modeling is particularly challenging because the system’s ionization states remains highly inhomogeneous and far from equilibrium during the X-ray probing time window. We can however use configuration average atomic calculations to qualitatively describe the trends of the observations. In Table 1 we present a calculation series of configuration average emission energies for Kα and Kβ transitions assuming three different initial atomic states, i.e. with six, three and zero 3d electrons present. Calculations were performed with the code of reference^20^. In agreement with the experimental data, these average emission energy calculations show a gradual shift of the Kα line toward lower energies when increasing the temperature of the system (as a lower number of 3d electrons is present). Differently, the energy of the Kβ lines after ionization is calculated to have a significantly larger energy shift, beyond the spectral window of the intact Κβ lines. Hence, their contribution would not affect the main centroid associated with the intact Kβ spectra but would instead manifest as a high energy tail. In addition, the dramatic reduction of the Kβ_2,5_ line within the first few fs shows that the conduction band electronic structure is extremely sensitive to the initial hot-electron cascades as the VTC spectral line vanishes well within electronic thermal equilibrium time scale of 10 s of fs. All these findings support the conclusion that there is a rapid redistribution of the valence electrons due to high-density photoelectron mediated secondary collisional ionization processes. Quantitative modeling of the Kβ_2,5_ spectra based on first principle predictions of valence electrons occupancy/character/localization for the dynamic and highly ionized conditions of this study is challenging. The data presented here can be particularly valuable for assessing these type of electronic structure models.

**Table 1:** Configuration average Hartree–Fock atomic calculations based Fe Kα and Kβ emission lines for three different initial Fe photo-excited states, i.e. with six, three and zero 3d electrons.

Initial State	Kα 1s^2^2p^5^ (eV)	Kβ 1s^2^3p^5^ (eV)
1s^1^3d^6^	6400.9	7058.0
1s^1^3d^3^	6399.1	7068.9
1s^1^3d^0^	6397.0	7089.1

Calculations were performed with the code of reference^20^.

The collisional ionization processes discussed above should present a strong concentration dependence, since the number of photoelectrons created is proportional to the number of dominant absorbing elements. At low concentrations, these absorbing elements are further apart, thus limiting the effects of the collisional ionizations. This should be corroborated by the observation of reduced spectral impact from measurements on more dilute solution phase samples. It is critical to understand the relative importance of spectral changes due to direct, multiple X-ray absorption by an atom, versus indirect changes due to collisional ionization, since many experiments in solution phase chemistry and biology have low concentrations of strong X-ray absorbers. We collected XES spectra from FeSO_4_ in aqueous solutions with 1.0 mol/L and 0.2 mol/L concentrations and compared the results to those of the pure metallic Fe sample (i.e., equivalent to an Fe concentration of ~ 140 mol/L). This difference in concentration corresponds to a factor of ~ 5.5 and ~ 8.9, respectively, larger average distance between individual Fe atoms as compared to the metallic sample.

**Figure 3:**
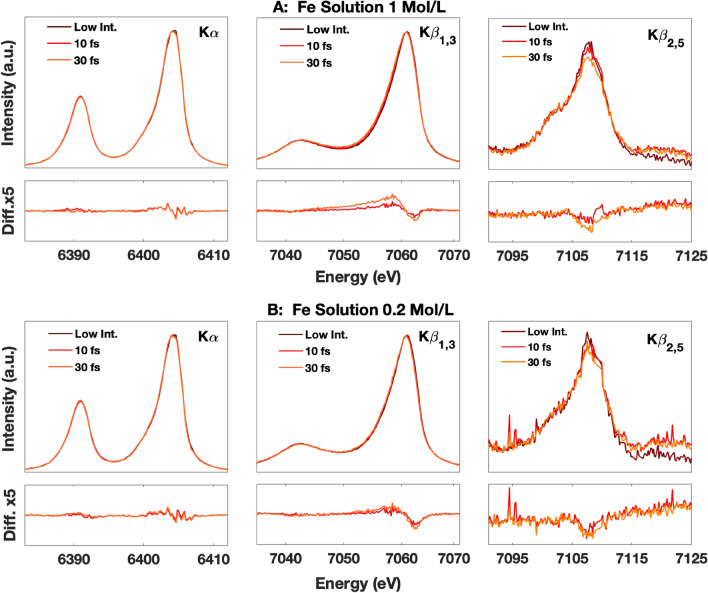
K emission spectra of FeSO_4_ with a concentration of 1 mol/L **(A)** and 0.2 mol/L **(B)** at different beam conditions (**A** and **B** TOP) and the difference spectra (with a 5 × zoom) compared to the unmodified spectrum acquired at low intensity (**A** and **B** BOTTOM). The 10 fs and 30 fs spectra were collected with a 2 μm size beam and intensities of 9 × 10^17^ and 5 × 10^17^ W cm^−2^ respectively.

Figure 3A,B show the Fe Kα, Kβ_1,3_ and Kβ_2,5_ of the 1.0 mol/L and 0.2 mol/L solutions respectively. The spectra, collected with the same X-ray conditions as for the metal foils, and pulse durations of 10 and 30 fs (~ 9 × 10^17^ W cm^−2^ and 5 × 10^17^ W cm^−2^ respectively) are compared with the unmodified spectrum acquired at low intensity. The direct comparison and the difference spectra show that the spectral changes in the Kα and Kβ spectra of the solution sample are much smaller than those of pure metallic Fe. The Kα spectral changes are insignificant while the Kβ spectral changes are relatively small but measurable. The 30 fs spectra show a concentration dependence with a larger change for the 1 mol/L sample compared to the 0.2 mol/L sample. The 10 fs spectra of the 1.0 mol/L and 0.2 mol/L samples show a similar change that is smaller than that of the 30 fs spectra. The 10 and 30 fs Kβ_2,5_ spectra of the 1.0 mol/L and 0.2 mol/L samples show an intensity loss as well, albeit significantly less than observed in solid Fe. The data statistics are unfortunately insufficient for contributing to a quantitative analysis of the differences between the two scenarios.

At approximately 0.45 mol/L, X-ray absorption becomes equal for water and the dissolved Fe ions. As the solute concentration is decreased to below that value, the overall absorption of the oxygen atoms starts to dominate. Therefore, most of the energy deposited in the sample is absorbed by the water molecules and the effect of the released electrons on the Fe XES will become concentration independent. For the measured concentration of 0.2 mol/L the absorption is dominated by water (75%), and we estimate that most of the observed changes can be attributed to electrons released from water ionization. The differences observed for the 0.2 mol/L solution sample therefore could be close to the lowest threshold of modifications attainable when using these X-ray beam conditions on solution samples. These observations further support the explanation based on the loss of outer electrons due to secondary collisional ionization processes.

Our study shows that observable X-ray induced modifications to the electronic structure of the samples occur even with sub-10 fs X-ray pulses, when the full intensity of the LCLS beam is focused to a micron scale spot size. Consequently, when designing an XFEL experiment, the selection of the incident beam parameters has to be carefully analyzed in order to avoid X-ray induced modifications in spectroscopic measurements. We note here that atomic structure probes may be less sensitive to the local electronic changes and may tolerate higher X-ray intensities before manifesting alterations in the data. We also note that this is not an issue for X-ray absorption (XAS) measurements using SASE pulses since it requires a monochromator that reduces the flux by a factor of ~ 100. Furthermore, laser pump X-ray probe measurements typically aim at measuring relative changes of spectra before and after photoexcitation. Therefore, the photo-induced changes obtained by subtracting a reference measured under the same XFEL conditions would thus be mostly insensitive to these processes. For experiments using “standard operating XFEL conditions” (e.g. 10 s μm size and ~ 30 fs pulse duration X-ray beam) to measure relevant samples (usually significantly below 0.2 mol/L concentration), the spectral modifications in time-resolved difference spectra are likely negligible, since the energy deposition in the sample is 2–3 orders of magnitude below the measurement conditions of the data shown on Fig. 3. On the other hand, for measurements of nonlinear X-ray processes, typically requiring very tight X-ray focus and short X-ray pulses to reach high intensities, some spectral changes due to the collisional processes and to the studied nonlinear processes could become hard to distinguish.

To summarize, we studied the fs-scale evolution of the K-shell X-ray emission spectra of Fe when illuminated with 10^17^–10^18^ W cm^−2^ intensity hard X-ray FEL pulses. For solid metallic Fe, we observed changes in the Fe K emission spectral shape as a function of X-ray intensity and pulse length (from 4 to 50 fs). For solution samples, where the photoabsorption/volume was significantly reduced, the observed changes were much smaller (i.e., 10–30 fs). We therefore attribute the observed spectral changes as mainly due to the high-density photo and Auger electron-induced secondary collisional ionization processes that ionize the valence electrons of the probed Fe atoms within the duration of the XFEL pulse. These spectral alterations have to be considered when designing XFEL experiments, in particular for concentrated samples and when the measurement requires long pulse lengths and/or strong focusing of the incident X-ray beam.

The majority of the time-resolved hard X-ray studies performed at XFELs use focal spot sizes larger than 10 μm and relatively dilute solutions with metal concentrations of less than 0.1 mol/L. Under these conditions, the changes in the XES spectra caused by X-ray induced modifications to the electronic structure are negligible. For experiments where the full XFEL beam is focused down well below 10 μm in diameter, the peak power becomes comparable to that used in this study (10^17^–10^18^ W cm^-2^). Under these conditions, the changes in the XES spectra caused by collisional ionization processes can be significant for highly concentrated samples. At low concentrations (< 0.1 mol/L) there are solvent ionization driven spectral changes that become concentration independent once the solvent absorption surpasses that of the metal ions. Depending on the required precision of the spectral analysis, these changes might have to be considered for electronic structure characterization. These observations are also relevant to further understanding the initial stages of the warm dense matter formation processes by XFELs. Specifically, contingent upon the existence of advanced models of temporally evolving spatial gradients of the incident radiation, they could help determining the various collision rates of high-energy electrons at solid densities. Our results might also contribute to benchmark incident XFEL X-ray pulse parameters for studies related to transition metal systems.

## Materials and methods

The XES spectra were measured at the XPP instrument^22^ of the Linac Coherent Light Source (LCLS) using the fundamental SASE radiation tuned to 8.2 keV at 120 Hz. Precise measurements of X-ray pulse energy, duration and focal spot sizes are essential for the accurate estimation of X-ray intensity at the sample. The shot-to-shot relative pulse energy was measured using a transmissive diagnostic^23^ and the average pulse length was characterized for each LCLS configuration using the X-ray transverse cavity diagnostic^24^. The relative X-ray pulse energy can be adjusted by inserting calibrated solid Si attenuators of different thickness upstream the sample. The X-ray pulse duration was varied from 4 to 50 fs by both adjusting the peak current of the XFEL as well as using a slotted spoiler foil in the electron bunch compressor^25^. The X-ray beam was focused using beryllium compound refractive lenses with a focal length of 4.0 m. The focal spot shape and size were characterized using both ptychography and imprint-based measurements, which confirmed a beam diameter of about ∼ 2.0 μm and good agreement with a Lorentzian spatial profile^26,27^. The absolute pulse energy at the sample was estimated based on both the imprint damage threshold and the beamline transmission calculation. The XES signal was detected with two energy dispersive von Hamos spectrometers using 500 mm radius crystal analyzers positioned at ~ 90 degrees with respect to the beam direction to minimize the scattering background^28^. Kα XES (using one Ge(440) analyzer) and Kβ_1,3_/Kβ’ or Kβ_2,5_ XES (using four Ge(620) analyzers) were recorded simultaneously, by two 2D position sensitive detectors, with an energy resolution of ≤ 1 eV. A schematic of the experimental setup can be found in reference^10^, Fig. 1^10^. All spectra shown in this letter are area normalized.

The samples used in this study include metallic Fe and aqueous Fe solutions. High purity metallic Fe thin films with a total thickness of 2.0 μm (1.0 μm Fe deposited, using electron beam evaporation, on each side of a 50 μm Kapton foil to provide sample flatness and rigidity) were raster scanned at 120 Hz (10 mm/s), so that each X-ray pulse probes a fresh sample location. Aqueous FeSO_4_ solutions with 1 mol/L and 0.2 mol/L Fe concentrations were delivered through a continuous flow of liquid sheet with a thickness of 100 μm at 45 degree with respect to the incident beam direction at a flow rate of about 6 m/s which also ensured full sample replenishment for each XFEL pulse.
